# Environmental DNA and RNA as Records of Human Exposome, Including Biotic/Abiotic Exposures and Its Implications in the Assessment of the Role of Environment in Chronic Diseases

**DOI:** 10.3390/ijms21144879

**Published:** 2020-07-10

**Authors:** Indu Shekhar Thakur, Deodutta Roy

**Affiliations:** 1 School of Environmental Sciences, Jawaharlal Nehru University, New Delhi 110067, India; 2 Department of Environmental Health Sciences, Florida International University, Miami, FL 33199, USA

**Keywords:** exposome, eDNA/eRNA, biomonitoring, health deficits

## Abstract

Most of environment-related diseases often result from multiple exposures of abiotic and/or biotic stressors across various life stages. The application of environmental DNA/RNA (eDNA/eRNA) to advance ecological understanding has been very successfully used. However, the eminent extension of eDNA/eRNA-based approaches to estimate human exposure to biotic and/or abiotic environmental stressors to understand the environmental causes of chronic diseases has yet to start. Here, we introduce the potential of eDNA/eRNA for bio-monitoring of human exposome and health effects in the real environmental or occupational settings. This review is the first of its kind to discuss how eDNA/eRNA-based approaches can be applied for assessing the human exposome. eDNA-based exposome assessment is expected to rely on our ability to capture the genome- and epigenome-wide signatures left behind by individuals in the indoor and outdoor physical spaces through shedding, excreting, etc. Records of eDNA/eRNA exposome may reflect the early appearance, persistence, and presence of biotic and/or abiotic-exposure-mediated modifications in these nucleic acid molecules. Functional genome- and epigenome-wide mapping of eDNA offer great promise to help elucidate the human exposome. Assessment of longitudinal exposure to physical, biological, and chemical agents present in the environment through eDNA/eRNA may enable the building of an integrative causal dynamic stochastic model to estimate environmental causes of human health deficits. This model is expected to incorporate key biological pathways and gene networks linking individuals, their geographic locations, and random multi-hits of environmental factors. Development and validation of monitoring of eDNA/eRNA exposome should seriously be considered to introduce into safety and risk assessment and as surrogates of chronic exposure to environmental stressors. Here we highlight that eDNA/eRNA reflecting longitudinal exposure of both biotic and abiotic environmental stressors may serve as records of human exposome and discuss its application as molecular tools for understanding the toxicogenomics basis of environment-related health deficits.

## 1. Introduction

**Figure 1:**
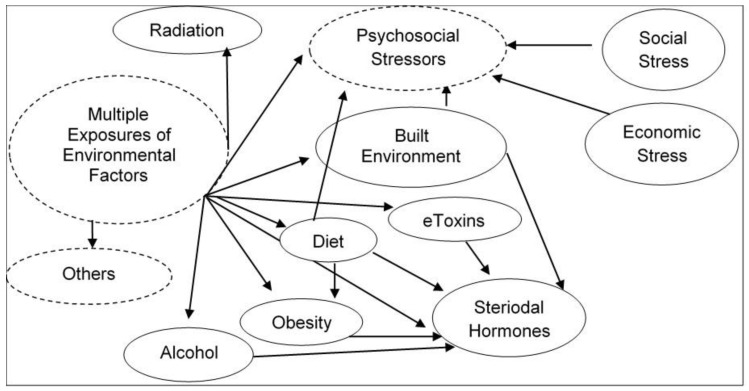
Illustration of complexity of cumulative multiple exposures of environmental stressors and their interactions in real-life human environmental settings.

Humans are stochastically exposed from conception/origin onwards to multiple sets of environmental factors present in the indoor and outdoor physical spaces where we live, recreate, and work. Exposure to these different sets of environments creates an overlapping human exposome. To clarify the environmental exposure overlaps, the exposome is categorized into three broad environmental domains [1]. The first environmental domain consists of a common external environment which may include the rural/urban environment (e.g., buildings, furnishings, open and public spaces, roads, utilities, and other infrastructure which either introduce, allow, or restrict environmental factors) and climate factors. This domain is followed with a specific external environment exposure in the indoor and outdoor physical spaces of specific toxins, pathogens, radiation, lifestyle factors (e.g., tobacco, alcohol), diet, physical activity, etc. Finally, the third domain consists of the internal environment resulting from the overall exposure of both common and specific environments. This domain may include internal biological factors such as metabolic factors, hormones, gut microflora, inflammation, oxidative stress, etc. These comprehensive and complete descriptions of dynamic environmental domains which individuals are exposed to on a regular basis indicate the complexity of cumulative multiple exposures of environmental stressors and their interactions in real-life human environmental settings (summarized in Figure 1). Exposures to these overlapping environmental external and internal factors are largely responsible for the majority of non-hereditary sporadic chronic diseases.

Comprehensive analyses of entire complex and dynamic environmental exposure to multiple stressors over time require high sampling effort and multi-species approaches [2]. Existing environmental exposure monitoring approaches are inefficient, not cost effective, destructive, and taxonomically or functionally biased. Also, there is a major reliance on professional taxonomic identification or organismal sample collections and analysis that are not translatable to real environmental settings. There is no fully validated and optimized tool available for mapping an entire exposome for an individual.

Accurate assessment of the exposome through estimating several hundreds of time-varying exposures has become a major challenge. High time resolution human exposome evaluation is currently being pursued using high-throughput omics technologies to integrate a wide range of exposures. However, the major hurdle for these approaches is accurately assessing the past exposure. The organismal genetic material, deoxyribonucleic acid (DNA), and ribonucleic acid (RNA) present in the environment (home, work or recreational built environment, hazardous wastes, lake and river water, air, etc.) refer to environmental DNA or RNA (eDNA/eRNA). All organisms, including microbes, animals, and humans leave behind traces of genetic material in the environment as they pass through habitats or interact with other organisms. Besides entire microorganism genomes (bacteria, virus, fungus and protozoans), the sources of eDNA/eRNA could be from human or animal blood, hair, excrements (urine, feces, mucous, saliva, secretions), skin, sperm, eggs, roots, leaves, fruit and pollen of plants, and rotting bodies of larger organisms. These genetic ‘breadcrumbs’ left behind, shed, or excreted from organisms, can serve as important biological materials for assessing the human exposome and health responses. This can be achieved by monitoring changes in the genomic and epigenomic marks in these eDNA/eRNA samples in relation to their environment [3]. The basis for this concept comes from the recent studies showing the genetic change through time recorded in the ancient DNA (aDNA), including sequence damage and reconstruction of temporal change in many large vertebrates from extreme climate cycles of paleoenvironments [4,5]. Like aDNA, the eDNA/eRNA are also to able keep records of long-lasting genetic and epigenetic changes from environmental insults and thus, they have potential to serve as a proxy of past and present exposure of multiple stressors. Previous eDNA monitoring has been mostly limited to the investigations related to studying the impact of the environment on biodiversity and ecological health [6]. Recently, there has been a rapid advancement in the application of eDNA/eRNA in biomonitoring of diseases, population genetics, and biosecurity [7]. Studies evaluating responses to environmental exposure to hazardous agents are mostly limited to monitoring areas with known pollutants or in controlled situations to determine differences in community composition. Study of how eDNA from microbes, plants, and animals that have changed and/or adapted over time can also serve as a record of exposure of environmental stressors over time. For example, ambient air, soil, rivers, and lakes, are continuously exposed to multiple stressors over time, including hazardous agent, anthropogenic activity, and changes in climate. There is an urgent need to analyze human eDNA to assess exposome effects on human health based on eDNA signatures observed in field conditions.

The applications of eDNA have been extensively reviewed [8,9,10]. However, to date, most of the eDNA reviews are focused on metagenome, ecological biodiversity, or rare species analyses. In this review, we introduce the new emerging application of eDNA in exposome assessment of human and other living organisms. This review is the first of its kind to discuss how eDNA/eRNA-based approaches can be applied for assessing human exposome. The combined eDNA/eRNA approach also holds promise for identifying genomic and epigenomic changes from emerging pathogens as well as other environmental biotic and abiotic stressors harming human health and for shedding light on the etiology of acute and chronic emerging health deficits.

## 2. eDNA and Environmental Impact Assessment

Hazardous outcomes caused by environmental stressors to ecological health are determined not only by toxic properties, but also changes in the ecosystems, including indigenous biodiversity and community composition. eDNA is one of the most emerging critical biological resources in the field of ecotoxicology [11]. Sequencing and bioinformatic analysis of eDNA samples have been extensively used to comprehensively investigate changes in the ecosystem and use it as a barometer of ecological health [10,12]. Therefore, here we briefly discuss it. Biodiversity monitoring is the standard for environmental impact assessment of anthropogenic activities. eDNA sequencing provides a powerful lens to detect alterations or modifications in biodiversity [13]. High-throughput sequencing (HTS) of eDNA metabarcoding of different ribosomal bacterial and eukaryotic markers is used to measure biotic index values, the taxonomic resolution of molecular markers, and measuring their performance to assess the environmental impact on biodiversity and metabolic function of numerous communities, including soils, ocean, human guts, oral cavity [14,15,16,17]. Most of the ecological and evolutionary metagenomics studies using sequencing of targeted marker genes, such as 16S/18S rDNA/rRNA, offer very limited knowledge about intraspecies diversity and no functional information. 

The eDNA has also been used for the detection of rare species, indicator species, or invasive species. This is accomplished by comparing a highly variable region as a barcode in the conserved regions in DNA among species to discriminate between species and determine the taxonomy. To determine various species, metabarcoding eDNA approaches and targeted assays have also been used on ice cores, dirt from swamps, archaeological sites, seawater, fecal samples, and herbal medicines. The application of eDNA metabarcoding extends across many applications. This has provided a huge new tool and more relevant approaches for assessments of risks posed to ecosystems by hazardous agents [11]. Researchers are additionally able to sample organisms and life stages which elude traditional techniques. For example, juvenile and gamete DNA are included in the detection of eDNA from water or sediments. eDNA analysis enables monitoring of the presence of the great crested newt—a protected species in Europe—and detecting invasive bighead carp in the Great Lakes, USA [18,19].

The efforts of studying air pollution are mostly focused on particulate matters in the ambient air, whereas inhalable microorganisms in polluted air have not been well investigated, and the few reported studies mostly focused on taxonomic identification rather than pathogenic potential of microbes. Next-generation sequencing (NGS) of eDNA is not only able to detect invasive species, pathogenic microbes, and responses to pollution, but it can also assess air quality, environmental exposure to chemicals, and their implications in human health [20,21,22,23]. For example, in the case of a major environment insults, such as leaching of toxins from hazardous waste sites, oil spill or intrusion of invasive species, eDNA sequencing and their analysis have been applied to detect what species have been impacted and what genomic changes have occurred in these species over time [24,25,26]. The presence of respiratory microbial allergens and pathogens in outdoors air samples from Beijing collected during a severe smog event and in hospital air samples have been detected by metabarcoding eDNA approaches [27,28]. In summary, these studies provide support, indicating that biodiversity detected by NGS of eDNA can also assess the health risk associated from exposure to environmental agents.

## 3. Can eDNA/eRNA Biomonitor the Exposome?

A single exposure to an internal or external environmental factor alone cannot explain the development of a complex chronic disease, rather it appears that exposure to multiple environmental factors across the lifespan and their interactions influence the development of a chronic disease in an individual (Figure 1). However, most of the experimental studies using cell lines and experiment models do not reflect nor mimic complex chronic exposure of hazards in real environmental or occupational settings. Traditional biomonitoring of exposome using DNA is limited to chemically modified adducts in blood, urine, or saliva samples [29]. The approach for untargeted biomonitoring of human exposome is being developed by combining genome-wide association study (GWAS), epigenome-wide association study (EWAS), and exposure-wide association study together; however, the development of this combined approach is in the very beginning stage [11,29]. Moreover, how the temporal and spatial environmental exposures that modulate the normal genetic/epigenetic and how phenotypic changes in a cell leading to the development of a particular type of disease phenotype will be assessed by these approaches remain to be sorted out.

$$\sum \frac{Environmental,\;Chemical,\;Physical\;\&\;Biological\;stressors}{Genomic\;and\;Epigenomic\;Modifications}\times Time=Environmental\;complex\;diseases$$

As depicted here in the summation scheme that it is a combined exposure of a multitude of environmental stressors over a period of time producing genomic and epigenomic modifications lead to the development and progression of complex environment-related diseases. This combined total exposure of chemical, physical, and biological stressors is not possible through the traditional DNA adduction approach limited to chemical exposome analysis in blood or urine samples, unable to capture exposure to biotic stressors. Untargeted biomonitoring approaches such as high-resolution metabolomics and liquid chromatography–high resolution mass spectrometers are limited to capturing exposure-related to exogenous and endogenous chemicals, abiotic stressors, and molecular risk factors [29]. Forensic environmental evidence uses samples of soil from clothing, a vehicle, or a crime scene—such as dust and environmental debris—for recovery of DNA that are leveraged to identify microbial and animal species present in that environment for the geolocation and individual identification [30,31]. The principal and application of eDNA in monitoring exposome is very similar to the DNA recovered/collected at crime scenes as a fingerprinting tool for forensic investigations. The eDNA can also discover if the target organism was/is present because each organism leaves a trail of DNA in their environmental habitats. Since eDNA can act as a forensic tool, the DNA extracted from environmental samples can provide the record of genomic and epigenomic modifications from the cumulative exposure of both biotic and abiotic stressors over time to all the living organisms present in the environmental settings—from the smallest bacteria to humans. However, genomic or epigenomic analysis of eDNA is limited to measure changes produced in the genetic materials by stressors. Whereas functional genomics and epigenomics approaches combine the performance of the transcriptomic analysis to measure expression patterns in the same cells tested for genome- and epigenome-wide changes of the same individuals. This is the reason, it is very important that a combined eDNA and eRNA approach is applied. This will help in the interpretation of causes and consequences of stressors-modified genetic and epigenetic signature(s) in eDNA/eRNA. Despite being an emerging new environmental monitoring technique, the enormous potential of eDNA application in biological monitoring has already been widely recognized and established [32]. Methods in eDNA research—particularly, sampling and handling of samples, DNA extraction, DNA half-life in aquatic, terrestrial and air samples, contamination, low abundance of DNA from higher organisms—have been extensively reviewed [32,33,34,35,36,37,38]. Therefore, herein we have not discussed these topics and related issues. The substantiation of the application of DNA isolated from environmental samples such as sewage, solid waste, dust, hair, water, or air in the indoor and outdoor physical spaces rather than directly collected from an individual living in that spaces can be a useful complement to conventional GWAS and EWAS methods and untargeted approaches for biomonitoring of human exposome.

One of the major arguments of critics of the use of eDNA/eRNA for biomonitoring human exposome has been their instability, a vast length of the genome/epigenome, and their inability to obtain individual gene specific structural and functional modifications associated with environmental stressors. Emerging evidence from NGS of eDNA of prehistoric sediments suggest that DNA shed from all organisms into their environments may be preserved for a long time [39]. Depending on the environmental conditions, the eDNA may persist for a varying length of time, ranging from hours to days and years. Unlike eDNA, RNA easily degrades in the environment, therefore eRNA has been less analyzed than eDNA as markers for environmental impacts. Changes detected in eRNA are expected to serve as a surrogate of ‘live’ signals in environmental samples [40]. Recent studies have shown the abundant amount of RNA persisting in the environment to measure gene expression [40,41]. The eRNA collected on biofilm matrix shows its prolonged persistence (21 days) [42]. There is some evidence showing that eRNA is more sensitive biomarker of environmental insults and may persist for a long period of time, particularly within extracellular vesicles or protein capsids [43]. Thus, eRNA present in sufficient amounts may allow investigation of real-time differential gene expression and splicing variation between exposed and non-exposed populations in real environmental settings. The functional analysis of the entire genome- and epigenome-mapping of eDNA/eRNA has great potential, not only for monitoring genomic and epigenomic modifications, but to genetically detect and identify emerging pathogens that may exert adverse effects at organismal/human levels. Most importantly, this method allows for biomonitoring without requiring collection of the living organism/samples from individuals, creating the ability to study organisms that are invasive, elusive, or endangered without introducing anthropogenic stress on the organism. The use of eDNA/eRNA compared to laboratory based genomic data presents several advantages for biomonitoring of hazardous site samples, contaminated freshwater and marine habitats, base line mapping of urban and rural cities, and residual effects of chronic exposure of environmental agents. For this, researchers collect air samples, water samples, soil/sediments, or other substrates, and isolate the DNA/RNA and sequence it with the latest powerful sequencing technologies. The sequences are then matched against reference libraries to assign species identities, and structural and numerical modifications in genomes/epigenomes compared to base line control eDNA/eRNA. It is very important to perform transcriptomic studies on the same environmental samples tested for epigenetic changes and genotyping of the collected samples from the same physical space. This will allow a number of causes and consequences of changes of epigenetic regulators to be interpreted. It is also critical that a longitudinal environmental molecular epidemiological design is implemented for repeated collection of eDNA/RNA over time, because this offers insights into temporal trends which is advantageous for both cumulative exposure biomarkers and environmental causal mechanistic information. Furthermore, the longitudinal sampling of eDNA/eRNA is expected to link prior and subsequent stressor exposures to the onset of a particular type of disease through the identification of the genome and epigenome modifications that may precede the appearance of an overt phenotype. 

## 4. Biomonitoring Exposome of Terrestrial, Fresh Water, and Marine Life

The eDNA analysis of genetic traces left behind by all plants, animals, and microbes is revolutionizing the understanding and management of environmental impacts on marine ecosystems through monitoring aquatic life in the coastal waters. The use of eDNA analysis of aquatic and terrestrial organisms provide information about not only the relationships between species, but also genomic and epigenomic patterns of each organism over space and time, and their responses to environmental change [43]. Longitudinal analysis of linkages of organisms ranging from microorganisms to mammals in a marine setting using high-throughput sequencing of multiple conserved genetic markers from eDNA showed dynamic relationships between marine ecosystem and environmental variables over time [44,45]. A recent study of exposure of heavy metals from anthropogenic sources on the DNA integrity showed that heavy metals and metalloid in the water from the polluted location produce genotoxic damages to a fish species, *Alburnus chalcoides* [46]. Chronic polluted coastal water exposure produced a decrease in DNA integrity coupled with a high degree of genotoxicity in clams [47]. Biomonitoring studies conducted in the vicinity of a coal power plant in Italy using the fish *Aphanius fasciatus* and the snail *Helix* sp. as sentinel organisms showed high level of heavy metal pollution accompanied by DNA damage over time. Fish from polluted sites compared to unpolluted sites show the highest levels of DNA damage compared to other two species [48]. These studies suggest that environmental stressors present in the real-life terrestrial, fresh water, and marine settings can be assessed by changes at the genome level of organisms and eDNA collected over time may reflect the record of footprints of a long-term exposure of environmental stressors.

## 5. Human Genome–Exposome Interface: Environmental Functional Genomics 

Most of the mutagenicity or genotoxicity investigations focus on the identification of mutations and copy number variation induced by environmental agents based on single genes. Next generation sequencing of human tumor genome shows distinct patterns of genomic alterations, including mutational spectra, involved in the causation of a specific-type of human tumor. Applying this whole genome sequencing, mutational spectra caused by environmental chemicals, such as benzo[a]pyrene, ultraviolet light, and aristolochic acid are also detected across the whole genome [49]. These mutagens induce a characteristic mutation signature: predominantly G→T mutations for BaP, C→T and CC→TT for UV and A→T for AA [49]. 

**Figure 2:**
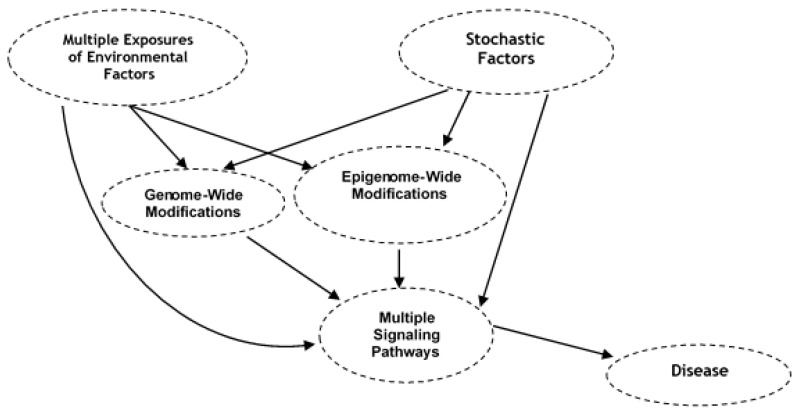
Scheme showing how eDNA record of genome- and epigenome-wide modifications of exposure to environmental stressors that may increase susceptibility of chronic diseases.

While there are challenges associated with eDNA/eRNA research, the field can profit from—and build upon—the framework of exoDNA technologies to overcome analysis of low abundant degraded highly heterogeneous multispecies eDNA. Rapid advances in single cell next-generation sequencing technologies and mutational analysis in cancer cells during the last decade have made possible to identify the environmentally induced genome-wide rare mutations and site-specific modifications in DNA. This is mostly accomplished thorough the Illumina next-generation genome-wide sequencing technique for double strand breaks (DSBs), the end-labeling procedure for single strand breaks (SSBs), the linear amplification/polymerase stop (LA/PS) assay for DSBs, SSBs, abasic sites and base damage, immunoprecipitation, and next generation sequencing (DDIP-seq), and rare damage and repair sequencing (RADAR-seq) [50,51]. The latter two techniques precisely locate DNA damage in the genome. Comprehensive RADAR-seq does not require DNA damage enrichment or amplification. A single microbial cell sequencing approach has been used to study the effects after an oil spill on the genomes of marine bacterioplankton. Similarly, advanced techniques—such as droplet microfluidics—facilitated capture and sequencing of eDNA from sloughed individual cells from fish, birds, reptiles, mammals, humans (isolated using flow cytometry and microfluidic technology), would allow obtaining individual genomic variation (changes in copy number, mutations) from eDNA samples of contaminated sites compared to the non-contaminated eDNA samples. It would also measure past accumulated and current exposure as well biological responses of exposure (effects) of contaminated hazardous agents to the community based on individual level genomic variation, because hazardous wastes are deposited over a long time at environmental land fill sites and could be a great genomics tools for environmental health risk assessment. The eDNA-based detection of disease-specific genomic alterations such as mutations, microsatellite alterations, and epigenetic modulations in circulating free DNA (cfDNA)—or quantitative changes in cfDNA, RNA, microRNAs (miRNAs), and exosomal DNA—offer highly promising approaches for the identification of eDNA causal gene signatures for risk of various diseases associated with exposure to environmental agents (Figure 2). NGS of eDNA approaches offer effective and efficient molecular tools to assess the hazard of environmental stressors, including anthropogenic pollutants on (1) the occurrences and population of wildlife, (2) emergence of drug resistant new invasive communities, and (3) the health of ecosystem and human.

While the development and validation of methods for the use of eDNA genome for exposome biomonitoring are yet to begin, the initiation of the development of this approach could tremendously benefit by building upon the advancements already made for recovery and sequencing of degraded, low levels of DNA in the fields of aDNA, environmental forensic DNA and eDNA-based population genetics monitoring [52,53,54]. Because, the eDNA/eRNA-based exposome assessment research would face some of the similar challenges as faced by these three fields of research to overcome the limits of detection as well as the complex matrix and mixture of different species DNA, and small mutations in a huge pool of non-mutated DNA. A silica-based DNA extraction produces the highest aDNA yields from highly degraded samples in less than ~15 min, with minimal carryover of contaminants that could inactivate the enzymes as well as inhibit library preparation for high-throughput sequencing [54]. Therefore, aDNA extraction protocol could be employed to prepare target DNA present in a low copy number in environmental sediment samples. Moreover, human and other mammal nuclear DNA appears to be less prone to degradation and damage perhaps over time due to better protection by wrapping of nuclear proteins around it and this enables the recovery of longer intact strands [55]. If the analysis of single nucleotide polymorphisms (SNP) can detect one SNP in exon or intron sequences and one 2-bp insertion–deletion polymorphism in exon or intron sequences in the Y-chromosomes specific gene(s) in low abundant and degraded human aDNA of multiple individuals and also contaminated with animal or plant or modern human ancient DNA [55], it is highly plausible for detecting a single mutation in a pool of non-mutated DNA recovered from environmental samples.

## 6. Epigenome Modifications from Exposure to Environmental Stressors that May Increase Susceptibility of Chronic Diseases

The epigenome is more sensitive to environmental stressors than the genome [56]. DNA methylation as an epigenetic readout in the genome has been used for monitoring and assessing the record of environmental exposure of chemical agents [57]. Epigenetic chemical modifications recorded in the eDNA resulting from environmental insults can be traced even after a long time of past exposure through detecting the modified epigenomes of the organisms. This can help in biomonitoring exposome and epigenomic signatures changing in response to environment stressors and disease phenotype. Several epidemiological studies have shown that famine, dietary restriction, and malnutrition cause low levels of methylation in DNA in children and this can be reversed by enriched diet and nutritional supplements including folate, zinc, and vitamins A, B, C, and D [58,59,60,61]. In addition to malnutrition, recently, epigenomic modifications from exposure to a large number of environmental agents have been documented and here we briefly discuss them. Prenatal exposure to an endocrine disruptor, bisphenol A, flame retardant chemicals, polybrominated diphenyl ethers, and tobacco smoking causes methylation in DNA sequences of genes [62,63,64,65]. Modifications in methylation at about 2,800 different positions on the entire genome of people affecting almost 400 genes who breathed in diesel fumes compared to people breathing the clean air have been reported [66]. Prenatal exposure to tobacco smoking to children from smoking mothers has been reported to alter methylation of various genes in cord blood and peripheral blood of their children at the age of 17 years. Methylation changes in genes may be associated with disease phenotype [67] or a phenotypic adaption to a particular stress. Therefore, it is critical to discern whether methylation changes in the genome arise as a result of direct exposure to environmental biotic and/or abiotic stressors or a phenotypic adaptation. To overcome this challenge, it is very important to characterize individual sample methylomes with a phenotype of individuals living in that physical space compared with appropriate controls. When a specific pattern of marks of genome-wide methylation is observed repeatedly at specific region(s) of the genome, discerning the phenotypic effects on the epigenome of eDNA from the physical space where control individuals live, and then only it may be considered as a proxy of environmental methylome perturbation occurring as a result of exposure to stressors. These epigenomic changes may be considered to be correlated, even possibly involved in the causal relationship with the phenotype. These findings suggest that past environmental exposure of a long period can be traced from modified DNA methylation in the genome. 

Residual effects of past exposure and effects of current exposure may also be possible to be detected by monitoring epigenetic modifications in transposable elements (TEs) [68]. TEs consisting of short or long repetitive sequences that were earlier considered as ‘junk DNA’, are now considered as driving forces of evolution and critical for gene regulatory functions. Epidemiological studies have shown that exposure to environmental stressors, including carcinogens, alters methylation and expression of TEs and initiate retro-transposition events. TEs are also very sensitive to the attack of other stressors, such as ionizing radiation (terrestrial, space, and UV-radiation), air pollutants, including particulate matter (PM)-derived and gaseous, and persistent organic hydrocarbons, and heavy metals [68,69]. These studies suggest that identification of eDNA record of epigenetic modifications in the methylome associated with exposure to environmental agents (Figure 2) that may increase susceptibility of chronic diseases could be potentially one of the most powerful biomonitoring approaches for exposome assessment.

## 7. Bioinformatics to Assess Causal Association between Environmental Exposure, Genome-Wide and Epigenome-Wide Modifications, and Disease Phenotype

**Figure 3:**
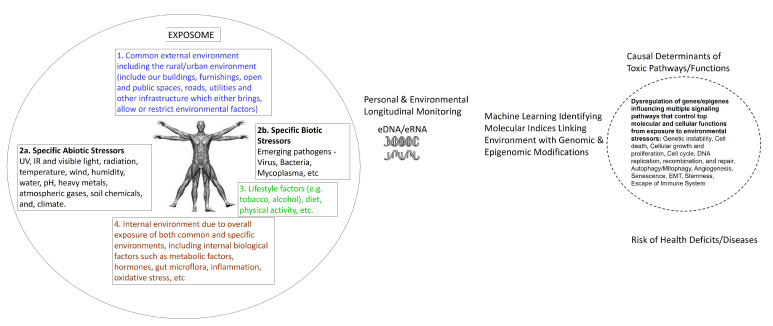
From the exposome-to-molecular toxicogenomics basis of assessing risk of environment related health deficits.

The step-wise guide for bioinformatics methods needed for taxonomic assignment are widely available through commercial sources, such as Illumina, Texagenomics as well as public online resources and has been extensively discussed [70,71,72,73]. The susceptibility to many human chronic diseases is considered to be result of the interactions of genome-wide changes, variations in epigenome and consequent dysregulation of multiple signaling pathways with environmental and stochastic factors during our life span—intrauterine to postnatal life, childhood, and adult life of an individual (Figure 2). Therefore, here we discuss bioinformatic methods to identify modified gene and epigene signatures in eDNA from time varying environmental exposure comparing to curated databases of control reference DNA sequences. Assessing the causal association between hundreds of time varying exposures producing modified genomic and epigenomic signatures and human health presents major statistical challenges. There is a lack of causal method(s) for the correlation structure of the exposome. Existing logistic regression or multivariate statistical analyses cannot fully and efficiently decipher the multiple exposures truly impacting the health deficits from correlated exposures. The random exposure of humans and other organisms to multiple environmental stressors over time call for the need for developing an integrative model. This type of the model could incorporate key biological pathways and networks and provide a more reliable estimate of human exposome and health deficits. As humans and other organisms interact with their indoor and outdoor physical spaces where we live, recreate, and work, cells left behind, shed, or excreted or waste in air, surface, or sewage contain the record of the total exposure in their DNA/RNA. Deep sequencing of these eDNA/eRNA are expected to provide both qualitative and quantitative estimates of about 21,306 protein-coding genes and 21,856 non-coding genes present in the human genome. Each environmental stressor is expected to produce a minimum of several hundred genome- and epigenome-wide modifications, and analyses of their interactions with the total environment will require billions of iterations. This type of large interaction can only be handled by machine learning methods [74,75]. Bioinformatics machine learning methods analyzing genome-wide modification and environmental data integration that links with disease phenotype can show the causal association between exposure of environmental endocrine disrupting chemicals and molecular risk factors that interact with these agents, and their association with the development of different disease phenotypes (Alzheimer’s, endometriosis, breast cancer, astrocytoma). Dysregulation of multiple signaling pathways that were influenced by environmental stressors in a particular disease was also uncovered [76,77,78]. Currently, there is also a lack of bioinformatics methods that can integrate diverse environmental, eDNA genomic and epigenomic, and epidemiological datasets into representations that can be interpreted in an exposome context. We employ the causal Bayesian network (CBN), a probabilistic graphical model, consisting of nodes of various random variables connected by links denoting causal influence. This method can establish a causal genetic and epigenetic network to understand the environmental causes of chronic diseases [2]. The Bayesian Markov chain Monte Carlo (MCMC) algorithmic search further helps to identify the temporal or longitudinal sequence or order of genetic and epigenetic changes and to establish the causality of these changes with a disease phenotype. These two statistical methods have also been extensively reviewed [5]. Here we introduce an integrated bioinformatics, and an eDNA epidemiological approach from the exposome to molecular toxicogenomics basis of assessing risk of environment related health deficits (Figure 3). The above two machine learning biostatistical methods are more well suited to accurately analyzing environmental insults recorded in eDNA/eRNA, characterizing a range of past and current exposure, and reconstructing functional relationships in human exposome. The feasibility of these bioinformatic approaches has been shown in the reconstruction of temporal environmental exposure to children resulting in genomic and proteomic changes that may cause cortical dysplasia and benign brain lesions [79,80]. Statistical machine learning graphical methods have previously been used to reconstruct temporal change in the genome of aDNA [4,5].

Human eDNA sequences may contain both cell-free DNA (cfDNA) and exosomal DNA (exoDNA) from blood and various body fluids such as saliva, mucus/sputum, breast milk, bile, and urine; and genomes (mitochondrial and nuclear) of exfoliated cells of feces and urine, gut and lung microbes, or epithelial shedding of skin, hair, and diet. Deep sequencing shows variation in double-stranded fragments of a low abundance short half-life and low purity exo DNA of disease cells [81,82,83,84,85]. Similar to exoDNA, environmental field DNA technological challenges include the recovery and sequencing of degraded, low copy number target DNA. Using above sensitive techniques, genomic alterations in extremely low abundance human eDNA from hazardous environmental waste sites should be detectable when compared to adjacent surrounding park, home, and garden environmental sample eDNA. The validity of this concept appears to be corroborated by personal and environmental longitudinal monitoring of dynamic human environmental exposome and examining its association with organismal or individual health deficits. This exposome study used longitudinal monitoring of personal airborne biotic and abiotic exposures, including microbial and viral, chemical, and environmental stressors and followed the personal exposomes of individuals of several distinct geographical locations. DNA and RNA exposomes showed that the DNA sequence mutations and variations in individuals located in the same general geographical region are diverse and dynamic, and these molecular risk factors changing as a function of age and environment may contribute significantly to human health deficits [11,86]. In summary, these studies suggest that the combined eDNA/eRNA approach analyzing both genomic and epigenomic modifications with exposure profiles may help to decipher the molecular susceptibility at the individual levels and assessing the risk of biological, chemical, physical, and spatial/lifestyle related health deficits.

## 8. Conclusions

Despite it being widely recognized that longitudinal multiple environmental exposures that modulate the genetic, epigenetic, and phenotypic changes contribute to chronic disease onset; we are not able to measure the total human exposure from our environment over time due to the lack of availability of tools to bio-monitor exposome. The records of eDNA exposome, including biotic and abiotic exposure that result in modifications in these nucleic acid molecules, have the potential to reflect their early appearance, persistence, and presence in both target organs and peripheral blood/excreta. Whereas eRNA may serve as a live record of exposome. With eDNA, versatile and sensitive multi-species approaches can be deployed, which generally have higher detection capabilities of detecting exposure and adverse impacts of environmental hazards. It is critical that we develop advanced molecular and bioinformatics technologies for monitoring of genomic/epigenomic alterations associated with real-life chronic and complex environmental exposures to hazards which would likely reveal an impact on eDNA/eRNA gene signatures, that can be used for monitoring, treating, and preventing environmentally related human health deficits. Monitoring of eDNA/eRNA exposome should seriously be considered for introduction into safety and risk assessment and as surrogates of chronic exposure to environmental stressors. There is already limited application of this approach in environmental field practice [87]. The further improvement of eDNA technologies has the potential to revolutionize biomonitoring of environmental exposure. Mining of functional environmental genomics/epigenomics data through constructing a high spatiotemporal database of eDNA using a non-targeted approach may become an innovative tool for the assessment of the human exposome in the future.
